# A Novel *SPAST* Mutation Results in Spastin Accumulation and Defects in Microtubule Dynamics

**DOI:** 10.1002/mds.28885

**Published:** 2021-12-20

**Authors:** Rui Chen, Shiyue Du, Yanyi Yao, Lu Zhang, Junyu Luo, Yinhua Shen, Zhenping Xu, Xiaomei Zeng, Luoying Zhang, Mugen Liu, Chuang Yin, Beisha Tang, Jun Tan, Xuan Xu, Jing Yu Liu

**Affiliations:** ^1^ College of Life Science and Technology Huazhong University of Science and Technology (HUST) Wuhan China; ^2^ Medical Genetics Center, Maternal and Child Health Hospital of Hubei Province Wuhan China; ^3^ College of Life Science and Technology Xinxiang Medical University Xinxiang China; ^4^ Department of Neurology Third Affiliated Hospital of Xinxiang Medical University Xinxiang China; ^5^ Department of Neurology Xiangya Hospital, Key Laboratory of Hunan Province in Neurodegenerative Disorders, Central South University Changsha China; ^6^ Institute of Neuroscience, State Key Laboratory of Neuroscience, Center for Excellence in Brain Science and Intelligence Technology, Chinese Academy of Sciences Shanghai China

**Keywords:** hereditary spastic paraplegias, *SPAST*, spastin, microtubule‐severing activity, microtubule dynamics

## Abstract

**Background:**

Haploinsufficiency is widely accepted as the pathogenic mechanism of spastic paraplegia type 4 (SPG4). However, there are some cases that cannot be explained by reduced function of the spastin protein encoded by *SPAST*.

**Objectives:**

To identify the causative gene of autosomal dominant hereditary spastic paraplegia in three large Chinese families and explore the pathological mechanism of a spastin variant.

**Methods:**

Three large Chinese hereditary spastic paraplegia families with a total of 247 individuals (67 patients) were investigated, of whom 59 members were recruited to the study. Genetic testing was performed to identify the causative gene. Western blotting and immunofluorescence were used to analyze the effects of the mutant proteins in vitro.

**Results:**

In the three hereditary spastic paraplegia families, of whom three index cases were misdiagnosed as other types of neurological diseases, a novel c.985dupA (p.Met329Asnfs*3) variant in *SPAST* was identified and was shown to cosegregate with the phenotype in the three families. The c.985dupA mutation produced two truncated mutants (mutant M1 and M87 isoforms) that accumulated to a higher level than their wild‐type counterparts. Furthermore, the mutant M1 isoform heavily decorated the microtubules and rendered them resistant to depolymerization. In contrast, the mutant M87 isoform was diffusely localized in both the nucleus and the cytoplasm, could not decorate microtubules, and was not able to promote microtubule disassembly.

**Conclusions:**

*SPAST* mutations leading to premature stop codons do not always act through haploinsufficiency. The truncated spastin may damage the corticospinal tracts through an isoform‐specific toxic effect.

## Graphical abstract

*SPAST* mRNA carrying the frameshift mutation c.985dupA was able to produce two stable truncated proteins (truncated M1 and M87 isoforms). The truncated spastin may damage the corticospinal tracts through an isoform‐specific pathological effect, thereby contributing to the pathogenesis of hereditary spastic paraplegia (HSP).

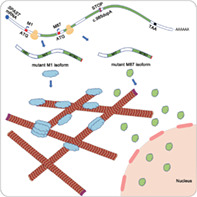

© 2021 The Authors. *Movement Disorders* published by Wiley Periodicals LLC on behalf of International Parkinson and Movement Disorder Society

Hereditary spastic paraplegia (HSP) is a genetically and clinically heterogeneous neurodegenerative disorder, and its main characteristics are progressive spasticity and weakness in the lower extremities.^1^ The overall estimated prevalence of HSP ranges from 1.27 to 9.8/100,000.^2^, ^3^, ^4^ HSPs are classified as ‘pure’ or ‘complicated’, depending on whether the patient also has other neurological manifestations, such as intellectual disability, optic neuropathy, epilepsy, ataxia, cognitive decline, extrapyramidal symptoms, and dysarthria, among others.^5^, ^6^, ^7^ Such wide clinical variability increases the difficulty of distinguishing this rare disease from other upper motor neuron diseases.

Currently, more than 79 loci and 65 genes have been defined in HSP patients,^8^ and all Mendelian modes of inheritance have been described.^9^ The proteins encoded by genes that cause HSP when mutated are involved in axonal path‐finding, membrane and axonal transport, endoplasmic reticulum morphogenesis, mitochondrial function, DNA repair, autophagy, lipid metabolism, and endosomal trafficking.^10^, ^11^ Although these are distinct cellular processes, most of them are microtubule‐based processes and are sensitive to perturbations of microtubule dynamics. Microtubules are critical for maintaining cell morphology, organelle transport, and cell motility in many cell types.^12^, ^13^ In the central nervous system, microtubules promote the growth of neuronal axons and maintain neurite complexity through their dynamic assembly and disassembly.^14^ Defects in microtubule dynamics are common features observed in many neurodegenerative diseases such as Alzheimer's disease, amyotrophic lateral sclerosis, and Huntington's disease.^15^

Spastic paraplegia type 4 (SPG4), which is caused by a pathogenic variant in *SPAST*, is the most common type of HSP typically with a pure phenotype.^2^, ^5^, ^16^ Spastin, the protein encoded by *SPAST*, is a microtubule‐severing ATPase enzyme that is involved in microtubule dynamics.^17^ The majority of pathogenic mutations in *SPAST* are nonsense, insertion, deletion, or splice‐site mutations, which are believed to reduce the amount of spastin protein as a result of the nonsense‐mediated decay of its mRNA.^16^, ^18^, ^19^ Therefore, inadequate microtubule severing resulting from haploinsufficiency has been proposed as the mechanism underlying HSP‐SPG4.^16^, ^18^ However, there is growing evidence that insufficient microtubule‐severing cannot fully explain the symptoms of HSP‐SPG4.^20^, ^21^, ^22^, ^23^ Abnormal aggregation of neurotoxic proteins is a major pathological feature of many neurodegenerative diseases.^24^, ^25^, ^26^ It has been reported that the cytotoxicity caused by intracellular accumulation of mutant spastin is detrimental to axon viability and transport.^27^, ^28^

In this study, we investigated three large families with pure HSP and identified a novel mutation (NM_014946.4: c.985dupA/p.Met329Asnfs*3) in *SPAST* through genetic analysis. The mutation c.985dupA did not promote mRNA decay but instead led to the production of two truncated mutants (mutant M1 and M87 isoforms), which were found to have a longer half‐life than the corresponding wild‐type isoforms and thus may be toxic to cells. Furthermore, we showed that the mutant M1 and M87 spastin isoforms exerted different effects on microtubule dynamics. Together, our findings support isoform‐specific pathological effects of truncated spastin, which may be an alternative pathological mechanism for HSP.

## 1 Subjects and Methods

### 1.1 Subjects and Genomic DNA Preparation

Three Chinese families with autosomal dominant HSPs were identified in three Hui villages in Henan Province. Peripheral blood (5 mL) was collected after informed consent was obtained from the participants, and genomic DNA was extracted from the peripheral blood samples as previously reported.^29^ All study protocols were approved by the Ethics Committee of Huazhong University of Science and Technology according to the Declaration of Helsinki. In addition to magnetic resonance imaging (MRI) scans of the brain and spinal cord, neurophysiological examinations including sensory nerve conduction studies, motor nerve conduction studies combined with F‐ware analysis, and somatosensory evoked potential studies were performed. Disease severity was assessed using the Spastic Paraplegia Rating Scale (SPRS).^30^

### 1.2 Mutation Identification

Linkage analysis was performed by using two microsatellite markers, *D2S165* and *D2S367*, which flank the *SPAST* gene. The pairwise logarithm of the odds (LOD) scores were calculated. Mutation analysis was performed via Sanger sequencing to identify the pathogenic variant. Primers were designed to amplify the exons of the *SPAST* gene, including all sequences at the junctions of exons and introns (Table S1). High‐resolution melting analysis was performed to detect the mutation in family members and 100 unrelated normal controls. For detailed methods refer to the Supplementary Materials (Appendix S1).

### 1.3 Plasmid Construction

The full‐length *SPAST* cDNA sequence was cloned into the pEGFP‐C1 expression vector, which expressed only the M1 spastin isoform. The plasmid carrying the mutation was generated based on the wild‐type plasmid using site‐directed mutagenesis (Vazyme) and verified by DNA sequencing. The M87 spastin‐expressing plasmid was obtained by deleting the N‐terminal part encoding the first 86 amino acids. The primer sequences are shown in Table S2.

### 1.4 Cell Culture and Transfection

HEK293 and neuro‐2A (N2A) cells were cultured in DMEM containing 10% fetal bovine serum (Gibco, Grand Island, NY, USA) and incubated at 37°C in 5% CO_2_, respectively. Transient transfection was performed using Lipofectamine 2000 (Invitrogen, Carlsbad, CA, USA).

### 1.5 Protein Stability Analysis

The cells were transfected with 1.5 μg plasmid, cultured for 24 hr and then treated with 10 μg/ml cycloheximide (CHX; APExBIO, Houston, TX, USA) for the indicated times (0, 4, 8, and 12 hr). The accumulated levels of wild‐type and mutated spastin isoforms were detected by Western blot analysis. Densitometry was performed using ImageJ software, and the average values were graphed. All experiments were performed three times independently.

### 1.6 Preparation of Cell Extracts

For the preparation of soluble and insoluble fractions, transfected cells were extracted with cold 0.1% Triton‐X‐100 in microtubule‐stabilizing buffer (0.1 M PIPES, pH 6.9, 2 mM EGTA, 1 mM MgSO_4_, and 0.1 mM EDTA) supplemented with protease inhibitor cocktail (cOmplete, Roche Diagnostic, Mannheim, Germany). The tubulin contents of soluble and insoluble fractions were measured by Western blot analysis.

### 1.7 Immunofluorescence

The cells were transfected and cultured for 24 hr, fixed with 10% formaldehyde for 15 min, and then treated with 0.5% Triton X‐100. After blocking with 5% Albumin Bovine V for 30 min, the cells were incubated with primary antibody (anti‐alpha tubulin: 1:200, AC012, ABclonal, China) overnight at 4°C and then incubated with a secondary antibody. The nuclei were stained with 4,6‐diamidino‐2‐phenylindole (DAPI). Images were captured using an FV1000 confocal microscope (Olympus, Tokyo, Japan).

### 1.8 Western Blotting

Protein samples were separated by 10% SDS‐PAGE and subsequently transferred to a nitrocellulose membrane. The membrane was then incubated with the indicated primary antibodies overnight at 4°C, and then reacted with goat anti‐mouse IgG coupled to horseradish peroxidase (HRP).

### 1.9 Statistical Analysis

Significant differences were determined by Student's t‐test and one‐way ANOVA. Data were considered statistically significant at *P* < 0.05 and presented as the mean ± standard error of the mean (SEM).

## 2 Results

### 2.1 Clinical Features

**FIG. 1:**
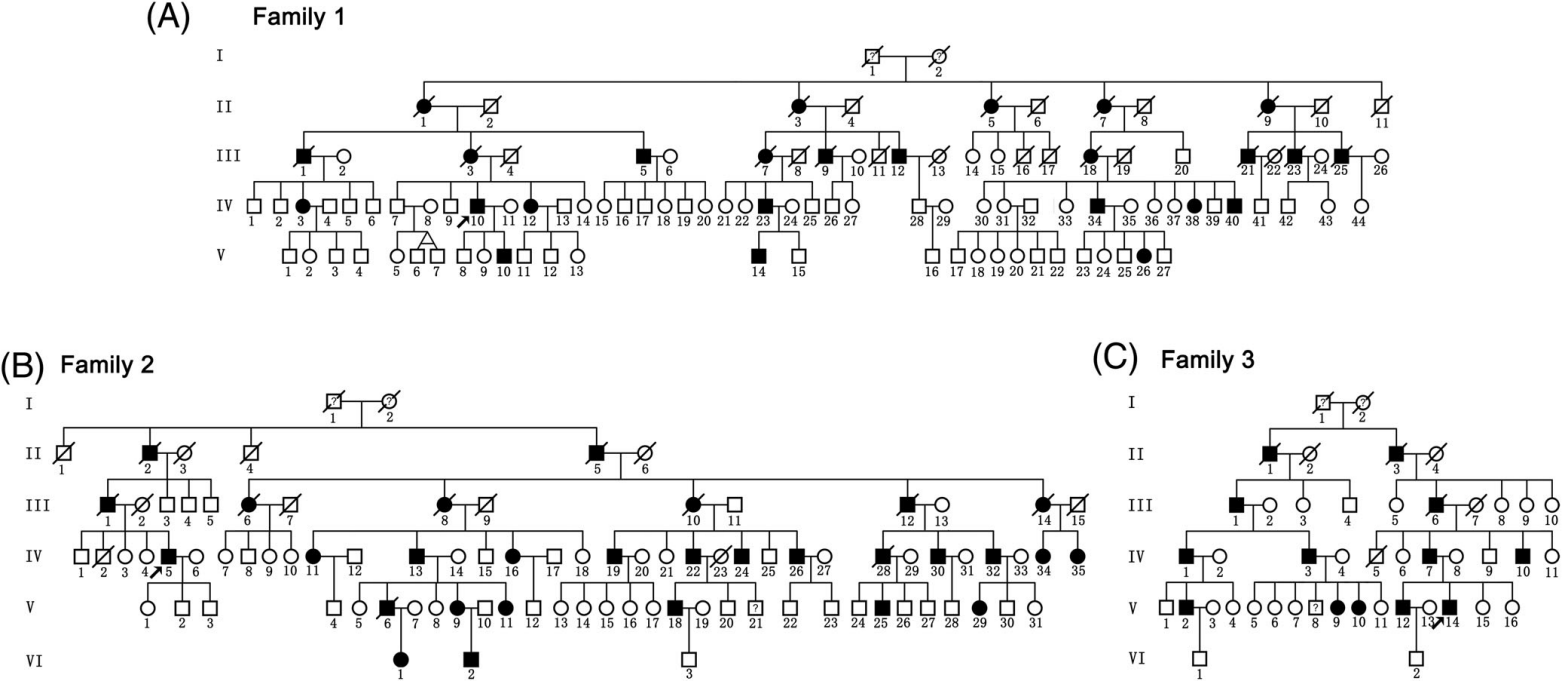
Pedigrees of three hereditary spastic paraparesis (HSP)‐affected families. (**A**), (**B**), (**C**) All pedigrees suggest autosomal dominant Mendelian inheritance. HSP‐affected individuals are marked by filled symbols; individuals with unclear disease status are marked by a question mark; the index case is marked with an arrow.

**FIG. 2:**
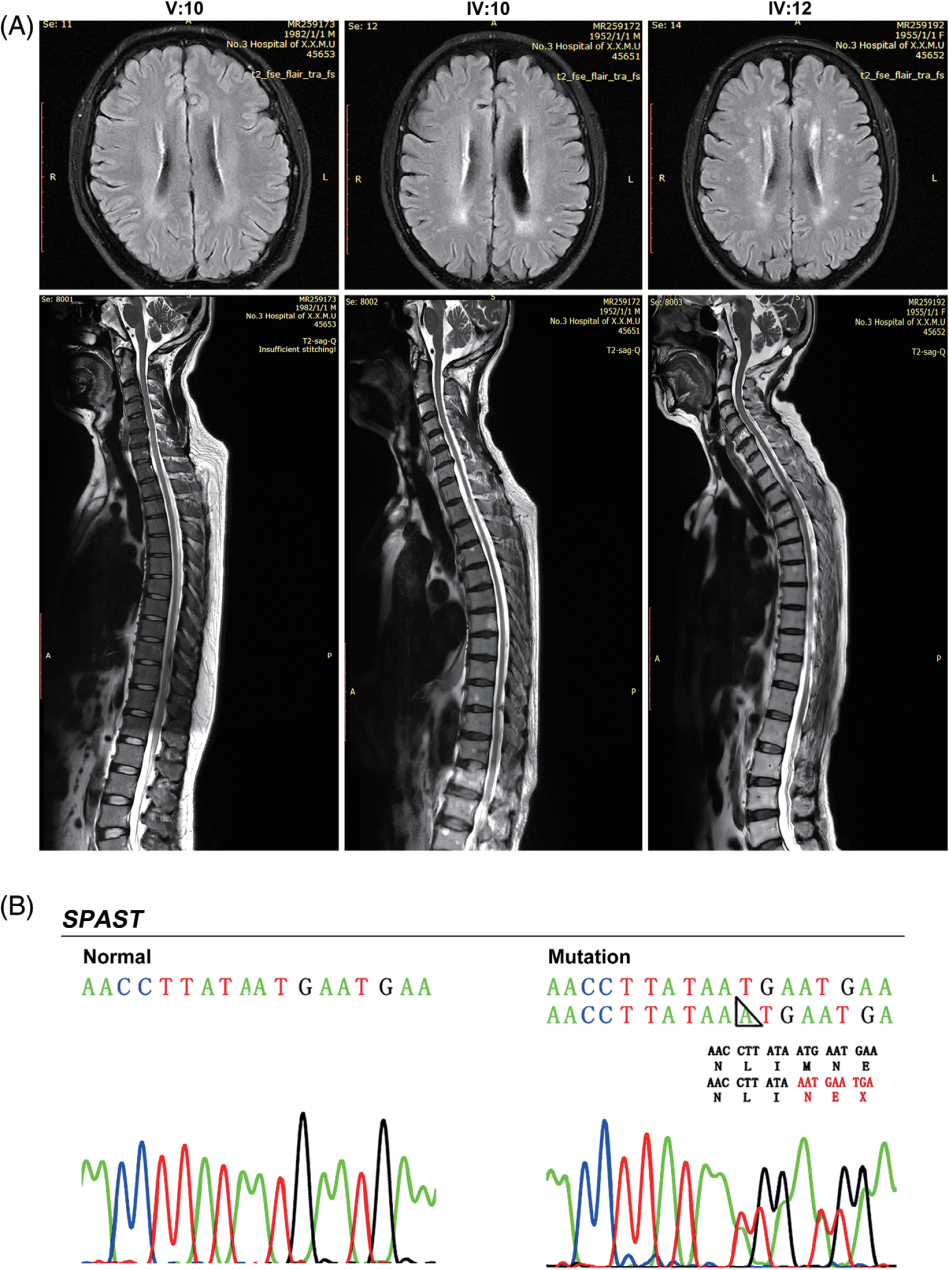
Magnetic resonance imaging (MRI) of the brain and spinal cord, and Sanger sequencing of the novel *SPAST* mutation. (**A**) T2‐weighted MRI of the brain (axial plane) and thoracic (sagittal plane) of patients V:10, IV:10, and IV:12 in Family 1. (**B**) The novel mutation was confirmed by Sanger sequencing. A black triangular box indicates the position of the *SPAST* mutations. The predicted amino acid sequence generated by the mutation is displayed below the wild‐type amino acid sequence. [Color figure can be viewed at wileyonlinelibrary.com]

Family 1 is a five‐generation family with 110 members (Fig. 1A), including 25 patients (13 males and 12 females). The index case (Fig. 1A, IV:10) developed lower limb weakness and stiffness around the age of 13 years. Subsequently, he experienced difficulty walking and upper body stiffness. He underwent selective posterior rhizotomy to relieve the symptoms of body stiffness at age 44 years. At present, the patient (69 years old) needs to use walking aids for long‐distance walking. He exhibited urinary dysfunction and had no abnormalities in intelligence. The neurological examination displayed muscle hypertonia, hyperactivity of the tendon reflex, Babinski sign, and scissor gait in both lower extremities. The total SPRS score was 37 points. The MRI scan showed T2 hyperintense signal changes in the periventricular white matter, indicating white matter lesions. The spinal cord was somewhat slender and suggestive of degenerative changes (Fig. 2A, IV:10). Neurophysiological examinations revealed decreased sensory conduction velocities of the median and ulnar nerves (Tables S3‐S5). Patient V:10 (39 years old) first noticed gait abnormalities around the age of 2 years, but the disease progressed very slowly in the following decades. Now the patient has only slight lower extremities stiffness, which does not affect exercise or walking. Neurological examination revealed ankle clonus and brisk bilateral knee reflexes. He had normal intelligence, and no urinary symptoms were observed. The SPRS score was 4 points. MRI scans of the brain and spinal cord as well as neurophysiological examinations showed no abnormalities (Fig. 2A and Tables S3‐S5). Patient IV:12 (66 years old) first noticed gait abnormalities around the age of 20 years and is now unable to walk. Neurological examination revealed muscle hypertonia and hyperactivity of the tendon reflex. The patient was of normal intelligence and had urinary symptoms. Neurophysiological examinations revealed decreased sensory conduction velocities of the median and ulnar nerves (Tables S3‐S5). The SPRS score was 47 points. Extensive T2 hyperintensity in the periventricular white matter areas and abnormal diffuse hyperintensities in the thoracic spinal cord along with thinning of the spinal cord were observed on T2‐weighted images (Fig. 2A, IV:12). Patient III:3 exhibited inability to walk upright since she was 60 years old and died at the age of 81 years. Patients IV:3, IV:38, and IV:40 exhibited only mild gait abnormalities.

Family 2 is a six‐generation family of 92 individuals (Fig. 1B) that includes 29 patients (17 males and 12 females). The index case (Fig. 1B, IV:5, 62 years old) exhibited progressive weakness, stiffness in the lower extremities, and difficulty walking beginning at 35 years of age. He was initially misdiagnosed with lateral sclerosis in the early disease stages. At present, the patient walks with assistive devices and has no abnormalities in intelligence. Patient IV:32 exhibited weakness of the lower extremities and clinical symptoms of scissor gait at 12–13 years of age. Patient V:29 began walking with a scissor gait at the age of 2 years. The clinical symptoms of other patients in the family were similar to those of the index case.

Family 3 is a six‐generation family with 45 members (Fig. 1C), among whom 13 patients (11 males and 2 females) were identified. The index case (Fig. 1C, V:14, 46 years old) developed symptoms of progressive weakness and stiffness in the lower extremities, with loss of mobility occurring at the age of 38 years. The index case (V:14) reported that he was misdiagnosed with syringomyelia in the early stage of the disease. At present, he is able to walk with the help of crutches. Other patients in this family have similar clinical symptoms.

### 2.2 Linkage Analysis

The results of the haplotype analysis are shown in Figure S1. All the patients in the three families carried the same polymorphism of marker *D2S165* except patients III:12 and IV:23 in Family 1 (Fig. S1A) and patient V:18 in Family 2 (Fig. S1B), who showed recombination events. However, only the marker *D2S367*, which is 2.05 Mb from *SPAST*, cosegregates with the disease in the three families (Fig. S1A‐C). Linkage analysis showed that the causal gene in the three families was linked to marker *D2S367* with LOD scores (θ = 0) of 6.48, 1.79, and 1.41. This evidence strongly suggested that *SPAST* was a common causative gene of the three HSP families in this study, and the linkage result also supported the conjecture that the three families shared a common ancestor.

### 2.3 Mutation Analysis

Sanger sequencing of the index case of the three families revealed a novel mutation c.985dupA in exon 6 of *SPAST* (Fig. 2B). The 1 base pair insertion caused a frameshift mutation that resulted in a premature termination codon at the 331st amino acid of SPAST, producing a truncated protein (p.Met329Asnfs*3) that completely lacks the AAA domain (Fig. 2B).

High‐resolution melting analysis was performed to detect whether the mutation cosegregated with the disease in the three families and whether the mutation was absent in 100 normal controls. We found that all patients in Family 1 had broader melting transition peaks (Fig. S2), indicating heterozygosity, while the normal samples had sharp melting transitions. The same result was obtained in Families 2 and 3, and member V:21 of Family 2 was also a heterozygous asymptomatic carrier (data not shown). Sharp melting transitions were obtained from 100 unrelated normal controls, showing that they were homozygous without insertion mutations (data not shown). According to the American College of Medical Genetics and Genomics (ACMG) criteria,^31^ this variant was evaluated as a pathogenic variant (PVS1 + PM2 + PP3). These results indicate that the mutation c.985dupA cosegregates with the disease, and is the genetic basis of the pathogenicity in the three families.

### 2.4 Effects of the SPAST c.985dupA Mutation on Spastin Protein Expression Level

**FIG. 3:**
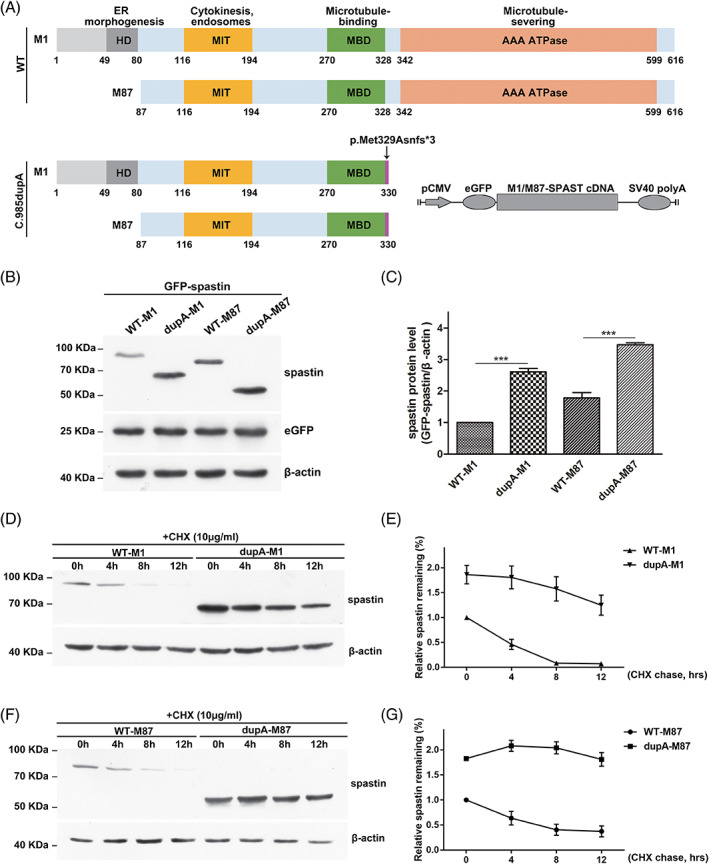
Characterization of c.985dupA *SPAST*. (**A**) Schematic structure of the human wild‐type (WT) and mutant spastin proteins. The black arrow indicates the location of the c.985dupA frameshift variant. M1, spastin isoforms (68 kDa); M87, spastin isoforms (60 kDa). Schematic of the spastin expression vectors is shown in the bottom right corner. (**B**) The protein expression of WT‐spastin (WT‐M1 and ‐M87) and c.985dupA‐spastin (dupA‐M1 and ‐M87). (**C**) Graphical representation of protein levels in (**B**) (n = 3, mean ± SEM, ***P < 0.001). The *P* values were calculated with Student's t‐test. (**D**) Time‐course stability analysis of mutant spastin (dupA‐M1). CHX, cycloheximide (10 μg/ml). (**E**) Statistical analysis of (**D**). All values were normalized to those of untreated controls. (**F**) Time‐course stability analysis of mutant spastin (dupA‐M87). (**G**) Statistical analysis of (**F**). [Color figure can be viewed at wileyonlinelibrary.com]

Reports have shown that other truncation mutations of the *SPAST* result in mRNA instability and reduced protein expression levels.^16^ *SPAST* mRNA has two initiation codons, which direct the synthesis of two distinct spastin isoforms, M1 (68 kDa) and M87 (60 kDa).^32^ Prediction of the stability of the RNA secondary structure by free energy minimization revealed no significant changes in the mutant mRNA (Fig. S3A, B). The mRNA expression levels of M1 and M87 were analyzed by transient expression of wild‐type or mutant spastin as a fusion protein with an enhanced green fluorescent protein (eGFP) at the amino‐terminus in HEK293 cells (eGFP tagged WT‐M1, c.985dupA‐M1, WT‐M87 or c.985dupA‐M87) (Fig. 3A). However, the expression of mutant mRNA did not change significantly (Fig. S3C), which indicated that the c.985dupA mutation had no significant effect on RNA stability. Then, protein expression levels of M1 and M87 were analyzed in HEK293 and N2A cells. Western blot analysis showed that the protein levels of the truncated eGFP‐tagged c.985dupA‐M1 (dupA‐M1) and c.985dupA‐M87 (dupA‐M87) were higher than those of their wild‐type counterparts (Fig. 3B, C and Fig. S4A, B). In addition, we evaluated the transfection efficiency by cotransfecting the pEGFP‐C1 vector, which confirmed that the increased truncated protein level was likely caused by increased protein stability instead of a difference in plasmid transfection efficiency (Fig. 3B).

To further verify this hypothesis, we performed a CHX (10 μg/ml) chase assay in HEK293 cells transiently expressing eGFP‐tagged wild‐type (WT‐M1 and WT‐M87) and mutant SPAST (dupA‐M1 and dupA‐M87) to inhibit de novo protein synthesis (Fig. 3D‐G). The wild‐type spastin protein (WT‐M1 and WT‐M87) synthesized before CHX treatment showed rapid degradation with increased CHX treatment time, while the two mutant proteins (dupA‐M1 and dupA‐M87) did not degrade significantly (Fig. 3D, F). Compared with wild‐type spastins, the truncated spastins accumulated in cells and had a longer half‐life (Fig. 3E, G), indicating that the c.985dupA mutation endowed the spastin protein isoforms with resistance to intracellular degradation.

### 2.5 Effects of c.985dupA on Spastin Localization and Microtubule Severing Activity

**FIG. 4:**
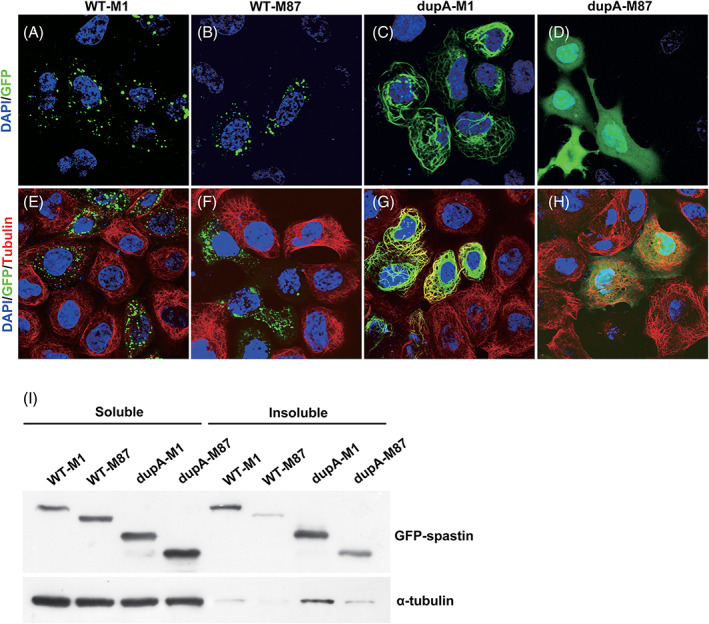
Effects of c.985dupA on spastin localization and microtubule integrity in HEK293 cells. (**A‐D**) Subcellular localization of green fluorescent protein (GFP)‐tagged spastin. (**E‐H**) Effects of c.985dupA on spastin microtubule‐severing activity. WT‐M1, wild‐type M1 isoform; WT‐M87, wild‐type M87 isoform; dupA‐M1, c.985dupA‐M1 isoform; dupA‐M87, c.985dupA‐M87 isoform. Representative immunofluorescence images for spastin (green), α‐tubulin (red), and nuclei (blue) were shown. Original magnification: 60× objective lens. (**I**) Effects of c.985dupA mutation on microtubule stability. The soluble and insoluble fractions of transfected cells were analyzed by Western blotting with the indicated antibodies. [Color figure can be viewed at wileyonlinelibrary.com]

Spastin is distributed in discrete punctate structures around the nucleus, and its correct positioning is essential for microtubule shearing.^17^, ^33^ To test whether the c.985dupA mutation altered the subcellular localization of spastin, eGFP‐tagged wild‐type and c.985dupA‐SPAST were transiently transfected into HEK293 and N2A cells. As previously reported,^33^ two wild‐type spastin isoforms (WT‐M1 and WT‐M87) showed a punctate expression pattern in cells (Fig. 4A, B and Fig. S4C). The mutant M1 isoform (dupA‐M1), which lacks the AAA domain, showed a strong filamentous expression pattern (Fig. 4C and Fig. S4C). Surprisingly, the mutant M87 isoform (dupA‐M87) exhibited a distinctive cellular localization (Fig. 4D and Fig. S4C). It was expressed in the cytoplasm and nucleus, suggesting that the physiological characteristics of truncated M87 changed significantly.

Next, microtubules in transfected HEK293 cells were stained with anti‐alpha tubulin antibody to investigate whether truncating mutations affected microtubule integrity. In our study, microtubules in cells transfected with WT‐M1 or WT‐M87 spastin were severed, and the microtubule‐severing activity of WT‐M1 was significantly lower than that of WT‐M87 (Fig. 4E, F), which was consistent with previous reports.^34^ Compared with neighbouring untransfected cells or wild‐type group, tubulin staining in dupA‐M87‐expressing cells appeared relatively normal (Fig. 4H), indicating that dupA‐M87 is defective in microtubule severing. In addition, we found that the spastin‐labeled filaments in dupA‐M1‐transfected cells colocalized with tubulin (Fig. 4G), that is, the microtubules were heavily decorated by mutant M1.

### 2.6 Effects of c.985dupA on Microtubule Stability

Cosedimentation assays were performed to ascertain whether truncating mutations affected microtubule stability in HEK293 cells. As shown in Figure 4I, a large fraction of dupA‐M1 cosedimented with the microtubules. In contrast, the amount of dupA‐M87 and microtubules recovered from the insoluble fractions were significantly greater than those in the WT‐M87 group but were not significantly different from those in the WT‐M1 group. In the WT‐M87 group, only a small amount of spastin and microtubules were detected in the insoluble fraction, and most of the spastin was recovered from the soluble fraction, which was consistent with the result in Figure 4A‐H. Although mutant M87 accumulates in cells, very little cosedimented with microtubules and it did not promote microtubule disassembly.

## 3 Discussion

Here we report three large Chinese families with HSP. There are 26 patients carrying a novel c.985dupA (p.Met329Asnfs*3) heterozygous mutation in *SPAST*, which might be inherited from a common ancestor. No additional clinical symptoms associated with complicated HSP were observed in any affected family members, and the disease was that of apparently pure HSP. The three patients in Family 1 who participated in further detailed examinations showed somehow different disease severity. Consistent with the SPRS scores, the MRI scan of patient IV:12 (score of 47) showed a sign of atrophy of the thoracic spinal cord, and the patient was unable to walk, whereas patient V:10 (score of 4) showed no signs of spinal cord atrophy, and the lower limbs were only subtly affected. Decreased sensory conduction velocities of the median and ulnar nerves in patients IV:10 and IV:12 indicated peripheral nerve involvement. This may be a consequence of the patient's condition worsening with age.^35^ Although white matter lesions have been reported to be associated with motor disability in SPG4 patients,^36^, ^37^ the white matter changes in patients IV:10 and IV:12 may be a physiological change that is common in older individuals without clinical significance.^38^ Therefore, long‐term follow‐up studies focusing on these patients are warranted.

The c.985dupA mutation did not affect *SPAST* mRNA stability but rather led to the production of two mutant isoforms with longer half‐lives than their wild‐type counterparts that consequently accumulated in cells (Fig. 3B‐G and Fig. S3C), which prompted us to consider the isoform‐specific toxic properties of c.985dupA mutant proteins. In fact, several spastin mutants with premature termination, such as c.734C > G (p.Ser245Ter) and c.550dupT (p.Asn184Ter), showed impaired microtubule‐severing activity, accumulated to notably higher levels than the wild‐type protein, and exhibited neurotoxicity in vitro.^34^ In addition, phenotypic studies in mouse and *Drosophila* models support a toxic gain‐of‐function mechanism of mutant spastin.^21^, ^39^ Given these findings, the abnormal accumulation of mutant spastin and the resulting cytotoxicity may contribute to the pathogenesis of HSP.^28^, ^34^ The p.Met329Asnfs*3 M87 (c.985dupA‐M87) was diffusely localized in both the nucleus and cytoplasm (Fig. 4D, H) and did not interfere with the depolymerization of microtubules (Fig. 4H). Considering that p.Met329Asnfs*3 M87 lacks the hydrophobic N‐terminus responsible for spastin assembly into a ring‐shaped hexamer (Fig. 3A), we speculate that the physiological characteristics of this abnormal protein have been altered significantly. The expression level of M87 in the spinal cord is much lower than that of M1,^28^ so even if p.Met329Asnfs*3 M87 is excessively stable, it may be relatively less harmful to the spinal cord.

The M1 isoform was detectable only in the adult spinal cord.^27^ The p.Met329Asnfs*3 M1 (c.985dupA‐M1), comprised of the N‐terminal region (amino acids 1–328) and two abnormal amino acids at the C‐terminus (Fig. 3A), also accumulated in cells and had a prolonged half‐life (Fig. 3D, E). Moreover, it can constitutively bind to microtubules (Fig. 4C, G), most likely through the microtubule binding domain (MTBD). This isoform cannot cut microtubules (Fig. 4G), so its long‐term occupation of microtubules interferes with normal microtubule dynamics. This may account for the observations that lesions in SPG4‐HSP patients are restricted to the cortical spinal tract and posterior column, and that patient condition worsens with age.

The isoform‐specific effects of the c.985dupA mutants M1 and M87 that we observed were based on the overexpressing cell systems, and this effect was a combined result of endogenous wild‐type spastin and excess exogenous mutant spastin. Since endogenous spastin protein expression is tightly regulated, we believe that the partial microtubule decoration by mutant M1 spastin could mimic what occurs in vivo. Actually, the preferential formation of axonal swellings between stable and dynamic microtubules in the distal axonal region has been described in an SPG4 animal model.^40^ Therefore, it would be inappropriate to explore in this model how the c.985dupA mutant affects the cellular processes closely related to axonal degeneration, such as intracellular transport and the distribution of organelles.^41^ A site‐specific model of the mutation, such as iPSC‐derived neurons or knock‐in mouse models, should allow this question to be addressed.

In nerve cells, the abolishment of spastin‐mediated microtubule severing resulted in defective synapse elimination and axonal transport, which are essential for neuronal differentiation and survival and rely on a dynamic microtubule cytoskeleton.^42^, ^43^, ^44^ Impaired mitochondrial transport was also observed in both SPG4 patients and animal models.^42^, ^45^, ^46^ These findings link axonal degeneration to axonal transport defects, which are associated with mutant spastin‐induced stabilization of microtubules. Our results indicate that mutant spastin exerts an isoform‐specific effect on microtubule dynamics, but we lack direct evidence of how dupA‐M1 and dupA‐M87 affect neuronal function and pathways relevant to SPG4 pathogenesis. Nonetheless, work from other teams has revealed the neuronal toxic effects of truncated spastin proteins.^27^, ^47^ These artificial mutant proteins completely lack the AAA domain and are nearly the same as the mutants found in our patient. In addition, data from our study (Fig. 4A‐H and Fig. S4C) and other teams suggest that the difference in the localization pattern of the two mutant isoforms was a manifestation of their molecular defects and did not vary with cell type.^21^, ^34^, ^47^ White et al. also showed that the MTBD domain (amino acids 270−328) mediates the filamentous localization pattern of truncation mutants.^17^ These published data can support our conclusion that truncated spastin, such as the c.985dupA/p.Met329Asnfs*3, may damage neuronal function through an isoform‐specific toxic effect. Further studies on the exact mechanism of its neural toxicity are certainly warranted.

In summary, we identified a novel *SPAST* mutation, c.985dupA, in three large Chinese HSP families, extending the known mutation spectrum of *SPAST*. Our study showed that reduced microtubule severing alone was not sufficient to account for the HSP‐SPG4 phenotypes caused by the c.985dupA mutation. Rather, the potential neurotoxicity to the corticospinal tract caused by the intracellular accumulation of truncated spastin should be considered as the pathogenesis of SPG4‐HSP.

## Author Roles

(1) Research Project: A. Conception, B. Organization, C. Execution; (2) Statistical Analysis: A. Design, B. Execution, C. Review and Critique; (3) Manuscript Preparation: A. Writing of the First Draft, B. Review and Critique.

R.C.: 1C, 2B, 3A

S.D.: 1C, 2B, 3A

Y.Y.: 1C, 3A

Lu Z.: 1C, 3B

J.L.: 1C, 3B

Y.S.:1C, 3B

Z.X.: 2B, 3B

X.Z.: 2C, 3B

Luoying Z.: 1C, 3B

M.L.: 1C, 3B

C.Y.: 1C, 3B

B.T.: 1C, 3B

J.T.: 1C, 3B

X.X.: 1B, 1C, 3B

J.Y.L.: 1A, 1B, 3B

## Full financial disclosures for the previous 12 months

Lu Z. has been employed with the Chinese Academy of Sciences (Institute of Neuroscience, Shanghai, China). Y.Y. is an associate chief physician at Maternal and Child Health Hospital (China) and has no financial disclosures. Z.X. is a researcher at the Xinxiang Medical University (China) and has no financial disclosures. M.L. is supported by the National Natural Science Foundation of China (Grant 81670890 and Grant 31871260). Luoying Z. is supported by the National Natural Science Foundation of China (Grant 31930021 and Grant 31671215). B.T. is supported by the State Key Program of National Natural Science Foundation of China (81130021). X.X. is performing his postdoctoral research at the Center for Excellence in Brain Science and Intelligence Technology, Chinese Academy of Sciences, and is supported by the Innovation Incentive Foundation (Center for Excellence in Brain Science and Intelligence Technology, Chinese Academy of Sciences, Shanghai, China). J.Y.L. has been employed by HUST and now works as a senior investigator in the Center for Excellence in Brain Science and Intelligence Technology, Chinese Academy of Sciences. Recently, she is supported by the following grants: National Natural Science Foundation of China (Grants 31871262 and 31671301) and by the National Key R&D Program of China (2016YFC1306000) and the Shanghai Municipal Science and Technology Major Project (2018SHZDZX05). The Shanghai Municipal Science and Technology Major Project (grant 2018SHZDZX05) was supported by the Science and Technology Commission of Shanghai Municipality. The National Key R&D Program of China (grant 2016YFC1306000) was supported by Ministry of Science and Technology of the People's Republic of China, which is equivalent to the National Natural Science Foundation of China. The other authors have nothing to disclose.

## Supporting information


**Appendix S1**. Supporting Information.

## Data Availability

The data that support the findings of this study are available from the corresponding author upon reasonable request.
