# Supplementary material for: A Novel SPAST Mutation Results in Spastin Accumulation and Defects in Microtubule Dynamics
Source: Mov Disord. 2021 Dec 20;37(3):598–607. doi: 10.1002/mds.28885 (PMC9300132; doi:10.1002/mds.28885)
Supplement: Supplementary file 1 — Appendix S1. Supporting Information. [file MDS-37-598-s001.docx]

**Supplementary Materials**

**Linkage analysis**

Two microsatellite markers that flank the *SPAST* gene, namely, *D2S165* and *D2S367*, were selected from the ABI PRISM Linkage Mapping set version 2.5. *D2S165* and *D2S367* are located 3.79 Mb upstream and 2.05 Mb downstream of *SPAST*, respectively. Pairwise logarithm of the odds (LOD) scores were calculated by using the Linkage Package 5.2 program assuming an autosomal dominant model.

**High-resolution melting (HRM) analysis**

HRM analysis to confirm the mutation was performed using a Rotor-Gene 6000 analyzer (QIAGEN). Briefly, the DNA fragment containing the mutation site was amplified, and syto9, a saturated fluorescent dye capable of insertion into double-stranded DNA, was incorporated during polymerase chain reaction (PCR). After amplification, the amplicons were subjected to melting from 76.05°C to 79.95°C at a ramp rate of 0.1°C/s. The results were analyzed using Rotor-Gene 6000 Series Software 1.7 (QIAGEN). The primer sequences used for the HRM analysis are shown in **Supplementary Table 6**.

**Detection of mRNA expression**

Total RNA extracted from HEK293 cells (transiently expressing eGFP-tagged WT and mutant SPAST) using TRIzol Reagent (TaKaRa Bio, Otsu, Japan) was reverse transcribed using a RevertAid First Strand cDNA Synthesis Kit (Thermo Fisher Scientific, Waltham, MA, USA) according to the manufacturers’ instructions. Semi-RT-PCR products were separated by electrophoresis on 1% agarose gels and visualized by ethidium bromide staining. Gene expression levels were normalized to *GAPDH*.

The primer sequences are shown in **Supplementary Table 7**.


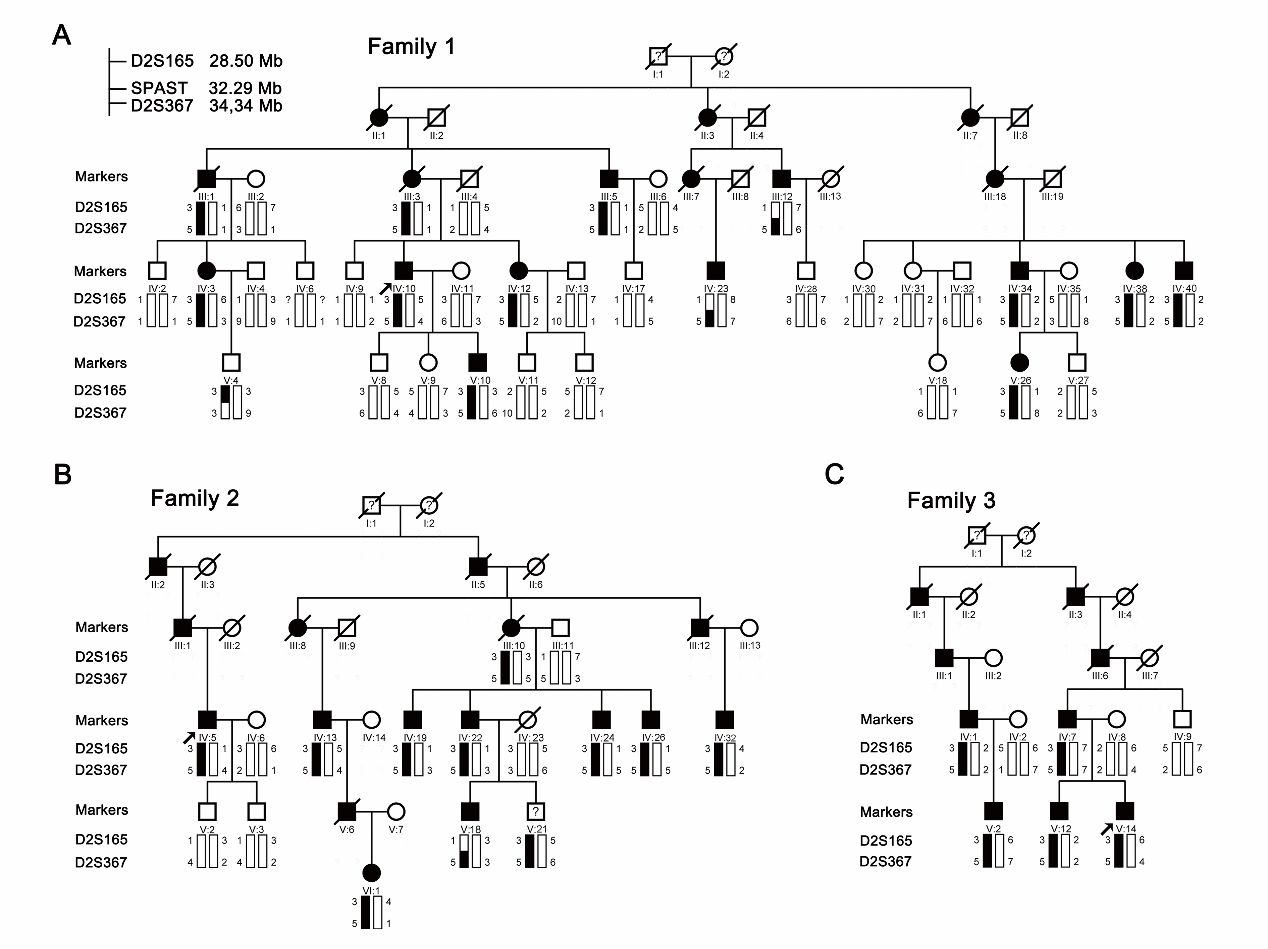


**Supplementary Figure 1.** Haplotype analysis of the three Chinese families for markers at the SPG4 locus. (A), (B), (C) Subjects were genotyped for two microsatellite markers, *D2S165* and *D2S367*, which flank the *SPAST* gene. Unavailable subjects were removed from the above analysis and are not shown on the pedigree.


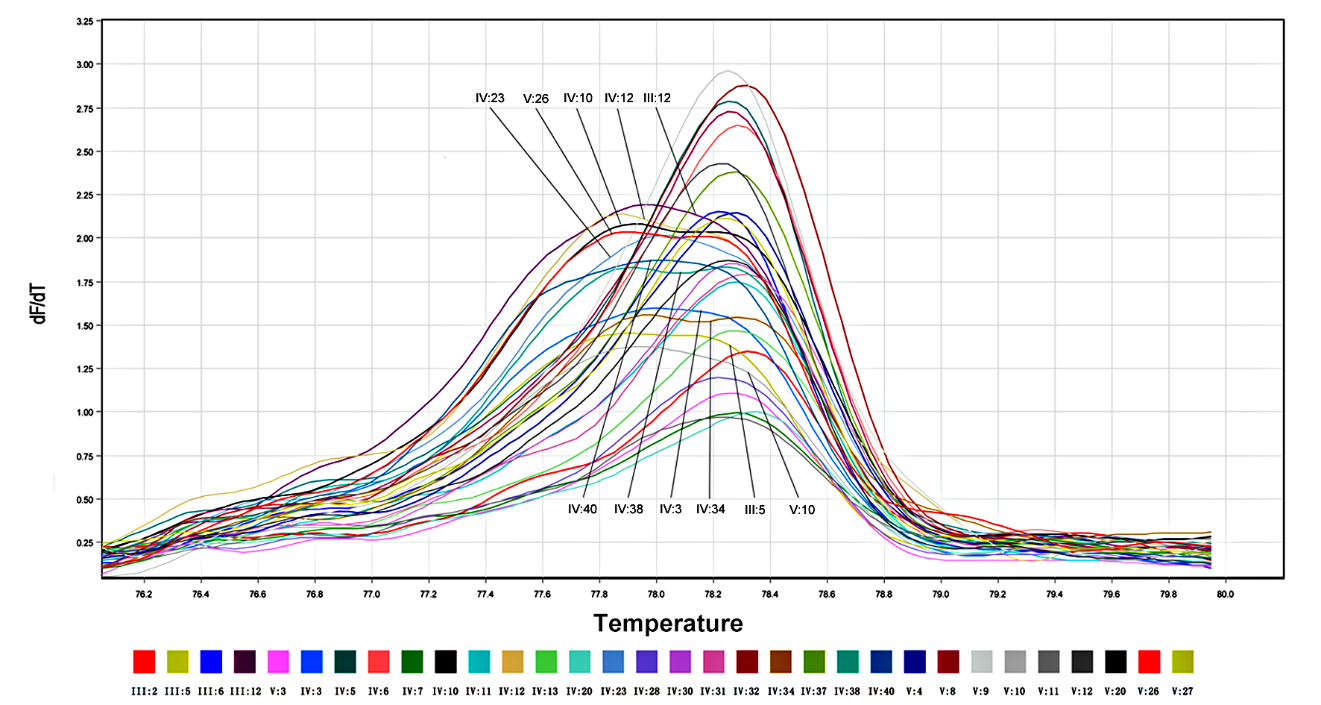


**Supplementary Figure 2.** HRM analysis in family 1. HRM analysis showing that all patients and the suspected patient V:26 in family 1 carry c.985dupA in SPAST. The curves represent the melting curves of the PCR products containing the mutation site from the 32 members in family 1. All patients (III:5, III:12, IV:3, IV:10, IV:12, IV:23, IV:34, IV:38, IV:40, V:10) and the suspected patient (V:26) marked in the figure have broader melting transition peaks, indicating that they are heterozygotes carrying the mutation. The unaffected samples have sharp melting transitions.


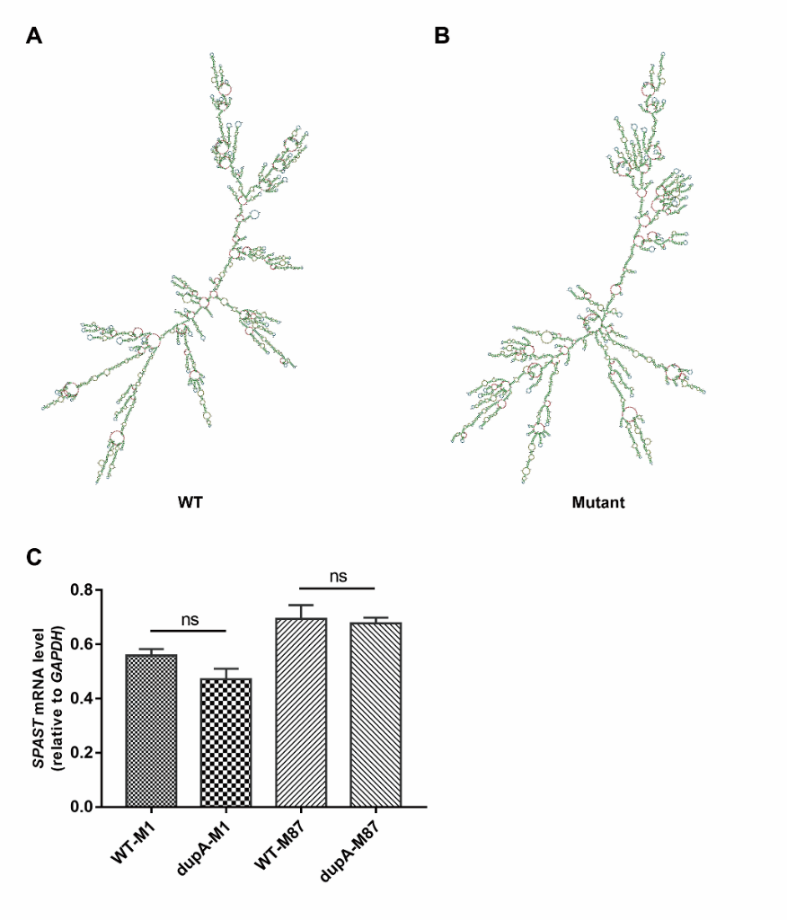


**Supplementary Figure 3.** Effects of the *SPAST* c.985dupA mutation on mRNA expression level. (A) MFE secondary structure of wild-type spastin mRNA. (B) MFE secondary structure of c.985dupA spastin mRNA. The stability of the RNA secondary structure was predicted using the minimum free energy (MFE) method (<http://rna.tbi.univie.ac.at/cgi-bin/RNAWebSuite/RNAfold.cgi>). The MFE values of the WT-*SPAST* RNA (A) and c.985dupA-*SPAST* RNA (B) secondary structures were -1571.22 kcal/mol and -1571.27 kcal/mol, respectively. (C) The expression level of WT- or c.985dupA *SPAST* mRNA in transfected HEK293 cells was detected by semiquantitative RT-PCR. The bar chart shows the mean ± SEM values from three replicates. Significant differences were determined by one-way ANOVA. The materials and methods are described in detail in the "**Detection of mRNA expression**" section in this document.


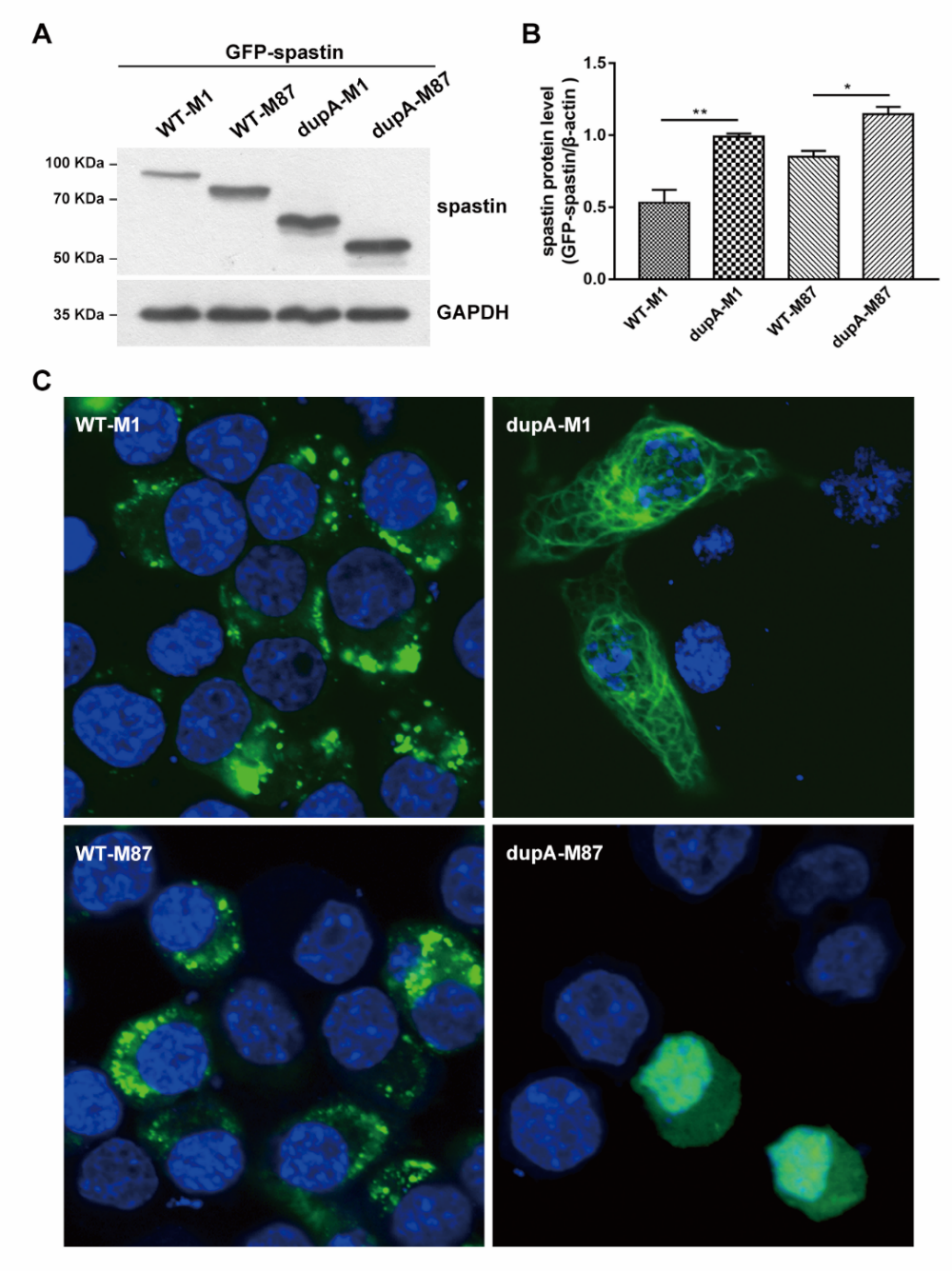


**Supplementary Figure 4.** Expression and subcellular localization patterns of mutant spastin isoforms in N2A cells. (A) The protein expression of WT-spastin (WT-M1 and M87) and c.985dupA-spastin (dupA-M1 and M87) in N2A cells. (B) Graphical representation of protein levels in (A) (n = 3, mean ± S.E.M., *P < 0.05; **P < 0.01). (C) Subcellular localization of GFP-tagged spastin in N2A cells. WT-M1, wild-type M1 isoform; WT-M87, wild-type M87 isoform; dupA-M1, c.985dupA-M1 isoform; dupA-M87, c.985dupA-M87 isoform. Representative immunofluorescence images for spastin (green), α-tubulin (red), and nuclei (blue) are shown. Original magnification: 60x objective lens.

**Supplementary Table** **1.** Primer sequences for pathogenic variant screening of *SPAST*

| Exon | Forward (5′to 3′) | | Reverse (5′ to 3′) | Annealing temperature °C |
| --- | --- | --- | --- | --- |
| 1 | | AGGAGGAGAAGGGGTTGTG | CGACCTACGGGAAAGCAGT | 63.2 ℃ |
| 2 | | TAAGGATCAATACCATGGGCTG | CCAGAAGTTTGAGGTTGCAGTG | 63.2 ℃ |
| 3 | | CCACAACACCTGGCCTAA | AGAGCAAGCGTCCATCTC | 57.4 ℃ |
| 4 | | CATGATGGTGCCACTGTACTT | GACTATACCTGACTTGTTGCATGT | 63.2 ℃ |
| 5 | | CTACCTAGTGACCACCCC | TAAGCAGGAATAGTATCTT | 57.3 ℃ |
| 6 | | AGTTTTGCTTACCCAGTCTGTT | GAAGCCTAAGCCCTATGACAT | 60.3 ℃ |
| 7 | | TAGGGCTTAGGCTTCATCTTG | CCTGGGCGACACAATGAGT | 60.3 ℃ |
| 8 | | GGCTCATGTAATCCTCCTGTCTT | CATGGCGAAATCCTGTCTCTG | 65.6 ℃ |
| 9 | | CCTGGCCTCATAGCTTACATTTT | AAGCCAGCCAGTTTACGGTATT | 60.3 ℃ |
| 10 | | GCACTTAACCAGGCTGTATGA | AGGCGTCTACTAGCATCGTG | 57.4 ℃ |
| 11 | | GTGGCTCGAGAACTTCAACCT | AAGCTCTTGTGGCCTATTAGTTG | 57.4 ℃ |
| 12 | | TGTGAAAGAAGAGAAGGGGAGCA | CATGAAGCCCAATAAAAGACGGT | 63.2 ℃ |
| 13 | | GCTTTTCCTGTCATTTGCTGTTT | TCTTAGGAGTGAAATTGCTGGCT | 57.4 ℃ |
| 14 | | GGATTGCTTGAACCCAGGATAT | AAGGCAAAGGAGGTAGAGGATG | 60.3 ℃ |
| 15 | | ACTTTGGGAGGCTGTGGC | TACTGGGAAGGCTGGGTG | 57.4 ℃ |
| 16 | | GCCCTTCAACAATTTCAACTGC | GCCGATATCATGCCAGACTGTC | 57.4 ℃ |
| 17 | | TCCATCATTTCGTTAACCACCAT | CCAGAATCAGGTTTTGTTGTGCT | 60.3 ℃ |

**Supplementary Table** **2.** Primers for constructing the eukaryotic expression vector of *SPAST*

| Primer | Sequence (5′ to 3′) |
| --- | --- |
| M1-KpnI-F | GGGGTACCATGAATTCTCCGGGTGGACGA |
| M1-BamHI-R | CGGGATCCTTAAACAGTGGTATCTCCAAAGTC |
| M87-KpnI-F | GGGGTACCATGGCAGCCAAGAGGAGCTC |
| M87-BamHI-R | CGGGATCCTTAAACAGTGGTATCTCCAAAGTC |
| Mut-Forward | GCTAACCTTATAAATGAATGAAATTGTGGA |
| Mut-Reverse | TCCACAATTTCATTCATTTATAAGGTTAG |

**Supplementary Table 3.** Somatosensory evoked potentials (SSEPs) of the median and tibial nerves in patients of family 1

|  | Lat/Amp (N20 wave) | |  | Lat/Amp (P40 wave) | |
| --- | --- | --- | --- | --- | --- |
| Subject | Left arm | Right arm |  | Left leg | Right leg |
| V:10 | 17.8/2.8 | 17.7/2.43 |  | 39.7/1.63 | 39.6/1.59 |
| IV:10 | 20.6/2.9 | 20.1/3.20 |  | 40.1/2.60 | 40.1/2.80 |
| IV:12 | 18.0/3.2 | 18.7/3.60 |  | 42.4/0.71 | 42.6/0.60 |

Median and tibial nerve SSEPs were acquired using surface electrodes over C3′/C4′ and Cz, respectively, and referred to Fz (International 10-20 system for electroencephalogram electrode placement). The lower-limb cortical response (P40 wave) and the upper-limb cortical response (N20 wave) were recorded. N20 (a negative, or upward, deflection at approximately 20 ms): cortical negativity of somatosensory potentials with median nerve stimulation. P40 (a positive, or downward, deflection at approximately 40 ms): cortical positivity of somatosensory potentials with tibial nerve stimulation. Amp: amplitude (mV); Lat: latency (ms).

**Supplementary Table 4.** Sensory nerve conduction studies in patients of family 1

|  | Subject | Nerve | Stimulation  site | Recording  site | Latency (ms) | |  | Amplitude (µV) | |  | velocity (m/s) | |  |
| --- | --- | --- | --- | --- | --- | --- | --- | --- | --- | --- | --- | --- | --- |
|  |  |  |  |  | Rt | Lt |  | Rt | Lt |  | Rt | Lt |  |
|  |  | Ulnar | Wrist | Fifth finger | 2.00 | 1.92 |  | 28.4 | 28.5 |  | 55.0 | 57.3 |  |
|  | V:10 | Median | Wrist | Second finger | 2.17 | 2.00 |  | 40.6 | 37.2 |  | 59.9 | 65.0 |  |
|  |  | Sural | Calf | Lateral  malleolus | 2.92 | 2.39 |  | 19.9 | 21.8 |  | 47.9 | 50.2 |  |
|  | | | | | | | | | | | | | |
|  |  | Ulnar | Wrist | Fifth finger | 2.73 | 2.24 |  | 7.1 | 13.8 |  | 36.6 | 44.6 |  |
|  | IV:10 | Median | Wrist | Second finger | 2.70 | 2.66 |  | 12.9 | 15.4 |  | 48.1 | 48.9 |  |
|  |  | Sural | Calf | Lateral  malleolus | 2.92 | 3.24 |  | 8.1 | 6.8 |  | 47.9 | 43.2 |  |
|  | | | | | | | | | | | | | |
|  |  | Ulnar | Wrist | Fifth finger | 2.14 | 2.46 |  | 24.4 | 36.4 |  | 46.7 | 40.7 |  |
|  | IV:12 | Median | Wrist | Second finger | 2.76 | 2.71 |  | 33.7 | 33.2 |  | 47.1 | 48.0 |  |
|  |  | Sural | Calf | Lateral  malleolus | 3.15 | 3.27 |  | 15.6 | 12.9 |  | 44.4 | 42.8 |  |

Rt, right; Lt, left;

**Supplementary Table 5.** Motor nerve conduction studies in patients of family 1

| Nerve | Stimulation site | Recording  site | | V:10 | | | |  | IV:10 | | | |  | IV:12 | | | |
| --- | --- | --- | --- | --- | --- | --- | --- | --- | --- | --- | --- | --- | --- | --- | --- | --- | --- |
|  |  |  |  | Lat | Amp | V | F-Lat |  | Lat | Amp | V | F-Lat |  | Lat | Amp | V | F-Lat |
| Ulnar | Wrist | ADM | Rt | 2.69 | 7.9 | - | 24.7 |  | 3.07 | 5.7 | - | 27.7 |  | 2.62 | 7.6 | - | 22.6 |
|  |  |  | Lt | 2.88 | 8.3 | - | 24.0 |  | 3.17 | 5.7 | - | 28.4 |  | 2.89 | 7.0 | - | 24.0 |
|  | Below elbow | Wrist | Rt | 4.91 | 7.8 | 63.1 | - |  | 5.88 | 5.1 | 53.4 | - |  | 4.54 | 7.6 | 62.5 | - |
|  |  |  | Lt | 5.23 | 8.1 | 63.8 | - |  | 5.92 | 5.7 | 54.5 | - |  | 5.10 | 6.8 | 54.3 | - |
|  | Above elbow- Below elbow | Wrist | Rt | 6.53 | 7.8 | 61.7 | - |  | 7.83 | 4.7 | 51.3 | - |  | 6.39 | 6.4 | 54.1 | - |
|  |  |  | Lt | 6.87 | 8.0 | 61.0 | - |  | 7.83 | 5.4 | 52.4 | - |  | 7.08 | 5.5 | 50.5 | - |
|  | | | | | | | | | | | | | | | | | |
| Median | Wrist | APB | Rt | 3.23 | 8.4 | - | 26.3 |  | 3.92 | 7.0 | - | 28.7 |  | 3.65 | 8.6 | - | 23.7 |
|  |  |  | Lt | 3.38 | 8.9 | - | 25.2 |  | 3.31 | 7.2 | - | 28.6 |  | 3.44 | 8.6 | - | 23.8 |
|  | Elbow | Wrist | Rt | 6.57 | 8.4 | 59.9 | - |  | 7.42 | 6.9 | 55.7 | - |  | 6.77 | 8.5 | 54.5 | - |
|  |  |  | Lt | 6.62 | 8.6 | 61.7 | - |  | 7.21 | 7.3 | 55.1 | - |  | 6.73 | 8.2 | 54.7 | - |

**continued**

| Nerve | Stimulation site | Recording  site | | V:10 | | | |  | IV:10 | | | |  | IV:12 | | | |
| --- | --- | --- | --- | --- | --- | --- | --- | --- | --- | --- | --- | --- | --- | --- | --- | --- | --- |
|  |  |  |  | Lat | Amp | V | F-Lat |  | Lat | Amp | V | F-Lat |  | Lat | Amp | V | F-Lat |
| Tibial | Ankle | AH | Rt | 4.00 | 11.6 | - | 46.6 |  | 6.46 | 2.9 | - | 49.9 |  | 4.51 | 10.6 | - | 45.1- |
|  |  |  | Lt | 3.81 | 9.1 | - | 45.0 |  | 6.05 | 0.81 | - | 48.5 |  | 5.98 | 6.8 | - | 43.4 |
|  | Popliteal fossa | Ankle | Rt | 11.7 | 9.3 | 57.1 | - |  | 14.3 | 1.90 | 46.6 | - |  | 11.2 | 7.5 | 50.8 | - |
|  |  |  | Lt | 11.6 | 7.0 | 52.6 | - |  | 12.6 | 0.47 | 49.7 | - |  | 13.0 | 4.7 | 47.6 | - |
|  | | | | | | | | | | | | | | | | | |
| Peroneal | Ankle | EDB | Rt | 3.93 | 5.7 | - | 45.9 |  | 4.14 | 1.51 | - | 45.5 |  | 3.75 | 3.1 | - | 42.5 |
|  |  |  | Lt | 3.21 | 7.5 | - | 42.9 |  | 3.73 | 2.1 | - | 45.8 |  | 3.47 | 1.07 | - | 41.5 |
|  | Below fibular | Ankle | Rt | 9.69 | 5.6 | 52.1 | - |  | 9.81 | 1.18 | 45.9 | - |  | 8.87 | 2.6 | 46.9 | - |
|  |  |  | Lt | 9.05 | 6.2 | 49.7 | - |  | 9.28 | 1.69 | 46.8 | - |  | 8.81 | 0.74 | 45.9 | - |
|  | Above fibular- Below fibular | Ankle | Rt | 10.8 | 5.5 | 49.5 | - |  | 11.5 | 1.12 | 44.4 | - |  | 10.3 | 2.5 | 42.0 | - |
|  |  |  | Lt | 10.2 | 5.8 | 47.8 | - |  | 10.7 | 1.66 | 42.3 | - |  | 10.6 | 0.55 | 44.7 | - |

Rt, right; Lt, left; Amp, amplitude (mV); CV, conduction velocity (m/s); F- Lat, F-wave latency (ms); ADM: abductor digiti minimi; EDB, extensor digitorum brevis; APB, abductor pollicis brevis; AH, abductor hallucis.

**Supplementary Table 6.** The primers for high-resolution melting (HRM) analysis

| primer | Sequence (5′ to 3′) |
| --- | --- |
| *SPAST*­_F | CTACCCCTACAACTGCTAC |
| *SPAST­*_R | TTTATTATCTATTTCACTCCTG |

**Supplementary Table 7.** The primers for detecting mRNA expression in HEK293 cells

| Primer | Sequence (5′ to 3′) |
| --- | --- |
| *SPAST*_SF | AGTCCGCCCTGAGCAAAGA |
| *SPAST*_SR | TACCGTCGACTGCAGAATTCGAAG |
| *GAPDH*_hF | AGATCCCTCCAAAATCAAGTGG |
| *GAPDH*_hR | GGCAGAGATGATGACCCTTTT |
